# Is Living in an Ethnic Enclave Associated With Better Cognitive Health of Older Immigrants? Results From PINE

**DOI:** 10.1093/geroni/igab046.193

**Published:** 2021-12-17

**Authors:** Man Guo, Yi Wang, Hanzhang Xu, Mengting Li, Bei Wu, XinQi Dong

**Affiliations:** 1 The University of Iowa, Iowa City, Iowa, United States; 2 University of Iowa, Iowa City, Iowa, United States; 3 Duke University School of Medicine, Duke University, North Carolina, United States; 4 Rutgers, The State University of New Jersey, New Brunswick, New Jersey, United States; 5 New York University, New York, New York, United States; 6 Rutgers University, Rutgers Institute for Health, New Jersey, United States

## Abstract

This study addressed three questions: 1) Is living in Chinatown associated with better cognition among Chinese older immigrants? 2) Is the association moderated by education, acculturation level, and social engagement? 3) Does the association vary by preferred language (Mandarin, Cantonese, Taishanese), an important indicator of heterogeneity among Chinese immigrants? Data were derived from the Population Study of Chinese Elderly in Chicago (N = 3,055). Results showed that Chinese older immigrants who lived in Chinatown had significantly poorer cognition than those who didn’t, and such a difference was largely due to educational differences between the two groups. Higher education or acculturation buffered the influence of Chinatown residence on cognitive health, but only among those who speak Mandarin. The findings indicate that living in an ethnic enclave may have a negative impact on cognitive function of Chinese older immigrants. The findings also reveal the sources of heterogeneity within the population.

